# CO_2_-dependent migration and relocation of LCIB, a pyrenoid-peripheral protein in *Chlamydomonas reinhardtii*

**DOI:** 10.1093/plphys/kiab528

**Published:** 2021-11-16

**Authors:** Takashi Yamano, Chihana Toyokawa, Daisuke Shimamura, Toshiki Matsuoka, Hideya Fukuzawa

**Affiliations:** Graduate School of Biostudies, Kyoto University, Kyoto 606-8502, Japan

## Abstract

Most microalgae overcome the difficulty of acquiring inorganic carbon (Ci) in aquatic environments by inducing a CO_2_-concentrating mechanism (CCM). In the green alga *Chlamydomonas reinhardtii*, two distinct photosynthetic acclimation states have been described under CO_2_-limiting conditions (low-CO_2_ [LC] and very low-CO_2_ [VLC]). LC-inducible protein B (LCIB), structurally characterized as carbonic anhydrase, localizes in the chloroplast stroma under CO_2_-supplied and LC conditions. In VLC conditions, it migrates to aggregate around the pyrenoid, where the CO_2_-fixing enzyme ribulose 1,5-bisphosphate carboxylase/oxygenase is enriched. Although the physiological importance of LCIB localization changes in the chloroplast has been shown, factors necessary for the localization changes remain uncertain. Here, we examined the effect of pH, light availability, photosynthetic electron flow, and protein synthesis on the localization changes, along with measuring Ci concentrations. LCIB dispersed or localized in the basal region of the chloroplast stroma at 8.3–15 µM CO_2_, whereas LCIB migrated toward the pyrenoid at 6.5 µM CO_2_. Furthermore, LCIB relocated toward the pyrenoid at 2.6–3.4 µM CO_2_, even in cells in the dark or treated with 3-(3,4-dichlorophenyl)-1,1-dimethylurea and cycloheximide in light. In contrast, in the mutant lacking *CCM1*, a master regulator of CCM, LCIB remained dispersed even at 4.3 µM CO_2_. Meanwhile, a simultaneous expression of LCIC, an interacting protein of LCIB, induced the localization of several speckled structures at the pyrenoid periphery. These results suggest that the localization changes of LCIB require LCIC and are controlled by CO_2_ concentration with ∼7 µM as the boundary.

## Teaser

Algal chloroplast proteins undergo localization changes in response to CO2 concentrations, reflecting their physiological function in survival under fluctuating CO2 environments.

## Introduction

CO_2_ fixation by photosynthesis in aquatic organisms is limited by several factors, including the slow diffusion rate of CO_2_ in the aquatic environment, the low catalytic activity of the CO_2_-fixing enzyme ribulose 1,5-bisphosphate carboxylase/oxygenase (Rubisco), and the oxygenase activity of Rubisco (Raven et al., 2011; Fukuzawa et al., 2012). To maintain CO_2_ fixation rates in such CO_2_-limited environments, most aquatic algae induce a CO_2_-concentrating mechanism (CCM) that actively takes up inorganic carbon (Ci; CO_2_ and ${\text{HCO}}_{3}^{–}$) into the cell and increases the CO_2_ concentration in the pyrenoid, a chloroplast microcompartment containing most of the cell’s Rubisco (Hennacy and Jonikas, 2020).

Pyrenoids are one of the central features of eukaryotic algal CCMs, increasing the CO_2_/O_2_ ratio at the active site of Rubisco, decreasing its oxygenase activity, and leading to maximal carboxylase activity. The molecular and biochemical aspects of pyrenoids have been well studied using the green alga *Chlamydomonas reinhardtii* (Meyer et al., 2017; Mackinder, 2018; Barrett et al., 2021). The *Chlamydomonas* pyrenoid is composed of a spherical Rubisco matrix that is traversed by pyrenoid tubules, a network of membranes continuous with stromal thylakoids (Ohad et al., 1967; Engel et al., 2015). Rubisco molecules are linked by essential pyrenoid component 1 (EPYC1; Mackinder et al., 2016). The Rubisco-EPYC1 system is dynamic and can undergo liquid–liquid phase separation, notably in response to changes in CO_2_ concentration (Freeman Rosenzweig et al., 2017; Wunder et al., 2018). A starch sheath, consisting of multiple starch plates, encapsulates the Rubisco-EPYC1 matrix to complete the pyrenoid structure (Sager and Palade, 1957).

Another essential feature of the CCM is its active Ci transport system that concentrates extracellular Ci in the form of CO_2_ into the pyrenoid. *Chlamydomonas* has at least two types of Ci transport systems depending on the CO_2_ concentration (Wang and Spalding, 2014a) and can adapt to at least two types of CO_2_-limiting conditions (Vance and Spalding 2005) called very low-CO_2_ (VLC) and low-CO_2_ (LC).

In VLC conditions, ${\text{HCO}}_{3}^{–}$ is actively transported from the extracellular medium to the chloroplast stroma by the cooperative function of high-light activated 3 (HLA3), an ABC-type transporter in the plasma membrane, and LC-inducible protein A (LCIA), an anion channel in the chloroplast envelope (Duanmu et al., 2009; Miura et al., 2004; Gao et al., 2015; Yamano et al., 2015). Furthermore, bestrophin-like proteins (BST1–3), anion channels in the thylakoid membrane, are thought to transport ${\text{HCO}}_{3}^{–}$ from the stroma to the thylakoid lumen (Mukherjee et al. 2019). Once in the lumen, carbonic anhydrase 3 (CAH3) conjugates with photosynthetic proton transport across the thylakoid membrane to convert ${\text{HCO}}_{3}^{–}$ to CO_2_, which diffuses into the pyrenoid matrix and is fixed by Rubisco (Karlsson et al., 1998).

In LC conditions, Ci-transport switches from the ${\text{HCO}}_{3}^{–}$ uptake system to a CO_2_ uptake system, and it has been proposed that the HLA3/LCIA-mediated ${\text{HCO}}_{3}^{–}$ uptake system is inhibited by an unknown mechanism as the CO_2_ concentration increases to LC levels (Wang and Spalding, 2014a). LCIB is an essential factor in the CO_2_ uptake system (Miura et al. 2004; Wang and Spalding, 2006); LCIB interacts with its homologous protein LCIC (Yamano et al., 2010), and the crystalline structures of these proteins resemble that of β-type carbonic anhydrase (CA), which possesses an active CA site that coordinates with a zinc ion (Jin et al., 2016). Based on these findings, the LCIB/LCIC complex is assumed to convert CO_2_ to ${\text{HCO}}_{3}^{–}$ to maintain the Ci pool in the chloroplast stroma.

LCIB accumulates slightly in high-CO_2_ (HC) conditions but is strongly accumulated in LC and VLC conditions (Yamano et al., 2010). The *lcib* mutant *ad1* cannot grow in LC conditions (air-dier phenotype; Wang and Spalding, 2006) but can survive in HC and VLC conditions. This unique phenotype is supported by a biphasic curve of photosynthetic O_2_-evolving activity of several *lcib* mutants. In VLC conditions, the O_2_-evolving activity of *lcib* mutants is comparable to that of wild-type (WT) cells, but it decreases in LC conditions, suggesting that LCIB is indispensable for survival in LC conditions (Yamano et al., 2010; Wang and Spalding, 2014a).

Of note, the localization of LCIB in the chloroplast changes in response to changes in CO_2_ concentration. In HC and LC conditions, LCIB is dispersed within the chloroplast stroma (Wang and Spalding, 2014a). In contrast, in VLC conditions, LCIB moves close to the pyrenoid to form the ring-like structure (referred to as “migration” in this study; Yamano et al., 2010; Wang and Spalding, 2014b). Furthermore, when the CO_2_ concentration is changed from VLC to HC or LC, LCIB disperses within the chloroplast stroma, but it again moves to around the pyrenoid when switching the CO_2_ concentrations from HC or LC to VLC (referred to as “relocation” in this study; Yamano et al., 2010). Recently, we showed that the structure of the starch sheath itself is required for LCIB localization around the pyrenoid and the maintenance of increased Ci-affinity in VLC conditions (Toyokawa et al., 2020). Thus, the physiological importance of LCIB function has been elucidated; however, the factors necessary and sufficient for LCIB migration and relocation are not fully understood.

In this study, to clarify the factors required for LCIB migration and relocation in the chloroplast, we traced the high-resolution localization of LCIB and examined the effect of pH, light availability, photosynthetic electron flow, and protein synthesis on the localization changes along with measuring Ci concentrations of the medium. We showed a series of evidence to support that the reversible localization changes of LCIB in the chloroplast was switched at an external CO_2_ concentration boundary of ∼7 µM without any requirement for light, photosynthetic electron flow, and de novo protein synthesis other than LCIC. Because LCIB is one of the key factors for driving the CCM, and homologs of LCIB are highly conserved in algal species harboring a CCM (Yamano et al., 2010), elucidating part of the mechanism of LCIB localization change will provide essential insights for CCM research.

## Results

### Isolation of a transgenic line expressing LCIB-Clover

**Figure 1:**
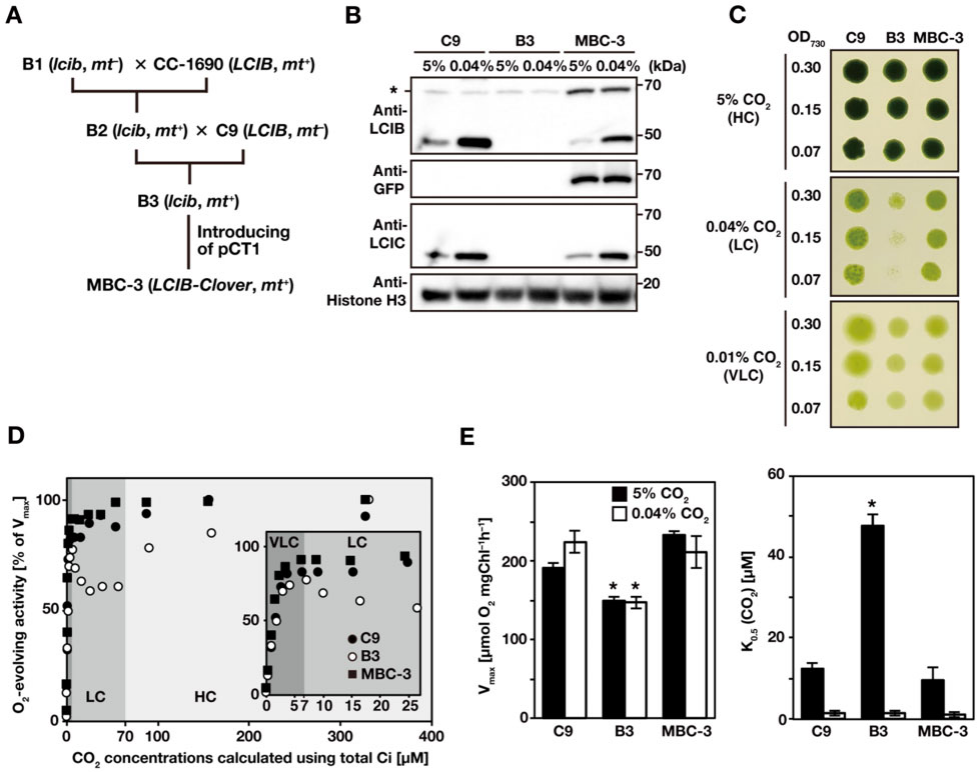
Isolation and characterization of LCIB-Clover expressing strain. **A,** Strategy to obtain the LCIB-Clover-expressing strain, MBC-3, by the genetic crossing of *lcib*-insertion mutant with WT strains (CC-1690 and C9) and introduction of the expression plasmid pCT1. *mt^–^*, mating type minus; *mt^+^*, mating type plus. **B,** Accumulation of LCIB, LCIC, and LCIB-Clover fusion proteins in C9, B3, and MBC-3 cells. Cells were grown in liquid culture aerated with 5% CO_2_ or 0.04% CO_2_ for 12 h. Histone H3 was used as a loading control. The asterisk indicates non-specific bands from C9 and B3 cells, which appeared at the same molecular weight as LCIB-Clover. kDa, kilo Dalton. **C,** Spot tests for the growth of C9, B3, and MBC-3 cells. Cell suspensions were diluted to the indicated optical density at 730 nm, and 3 µL of each diluted cell suspension was spotted onto MOPS-P agar plates and incubated in HC (5% CO_2_) conditions for 4 d, LC (0.04% CO_2_) conditions for 5 d, or VLC (0.01% CO_2_) conditions for 8 d. **D,** Typical responses of net O_2_-evolving activities of C9 (closed circles), B3 (open circles), and MBC-3 cells (closed squares) against calculated CO_2_ concentrations at pH 7.8 for the ranges of 0–400 µM CO_2_ and 0–27.5 µM CO_2_ (inset). Shaded areas represent different physiological CO_2_-acclimation states of *Chlamydomonas*: VLC (<7 µM CO_2_; dark gray), LC (7–70 µM; medium gray), and HC (>70 µM CO_2_; light gray). CO_2_ concentrations were calculated from the concentration of Ci added, assuming that CO_2_ and ${\text{HCO}}_{3}^{–}$ are in equilibrium due to the activity of CA localized in the periplasmic space. Before measurements, cells were grown in the liquid culture aerated with 0.04% CO_2_ for 12 h. **E,** V_max_ and K_0.5_ (CO_2_) values. The CO_2_ concentration required for the half-maximal rate of V_max_ of C9, B3, and MBC-3 cells was calculated from the O_2_-evolving activities in D. Data in all experiments are mean values ± standard deviation from three or four biological replicates. *P-value < 0.01, Student’s *t* test.

In our previous study (Toyokawa et al., 2020), we used *lcib*-insertion mutant B1 obtained from the *Chlamydomonas* Library Project (CLiP; Li et al., 2016). However, B1 cells had a lower amount of chlorophyll per cell, smaller cell size, and adhered to the test tube surface more than the WT strain C9, making it unsuitable for physiological experiments. Thus, to isolate an *lcib*-insertion mutant with a C9 background, we first crossed B1 with WT strain CC-1690 (*LCIB*, *mt*^+^) and obtained progeny strain F_1_ line 73-4 (*lcib*, *mt^+^*; B2 hereafter; Figure  1A). Next, we crossed B2 with C9 (*LCIB*, *mt*^–^) and obtained five progenies. Among these, strain F_2_ 99-4 (*lcib*, *mt^+^*; B3 hereafter) showed a comparable amount of chlorophyll per cell and cell size to C9 cells. Finally, to visualize the localization of LCIB in vivo, we introduced an LCIB-Clover expression plasmid (pCT1; Nitta et al., 2018), in which the target gene is driven by a constitutive *HSP70A*-*RBCS2* promoter and terminated by *RBCS2* 3′ -UTR, into B3 cells and obtained strain MBC-3 (*LCIB-Clover*, *mt^+^*). In the MBC-3 cells, the fluorescence signal of Clover (a green fluorescence protein variant) fused with LCIB was observed around the pyrenoid in VLC conditions. In B3 cells, LCIC (49 kDa), the interacting protein of LCIB (48 kDa), was also hardly detected as with the previously reported *LCIB* RNAi strains (Yamano et al., 2010); however, the LCIC accumulation was recovered in MBC-3 cells along with the accumulation of LCIB-Clover (68 kDa; Figure  1B).

To examine whether LCIB-Clover in MBC-3 cells was functional in vivo, we compared the growth rates of C9, B3, and MBC-3 cells in different CO_2_ conditions and evaluated their photosynthetic characteristics by measuring their Ci-dependent O_2_-evolving activity. The growth of B3 cells was inhibited in LC conditions but not in HC and VLC conditions (Figure  1C). Ci-depleted B3 cells grown in medium aerated with 0.04% CO_2_ showed a biphasic curve of photosynthetic O_2_-evolving activity with the gradual addition of NaHCO_3_ from lower to higher concentrations (Figure  1D); the activity increased at calculated CO_2_ concentrations <7 µM, decreased between 7 and 70 µM CO_2_, and again increased above a concentration of ∼70 µM CO_2_. The CO_2_ concentration of 7 µM, at which the O_2_-evolving activity of B3 cells switched from increasing to decreasing, corresponded to the threshold CO_2_ concentrations in VLC and LC conditions as reported previously (Wang and Spalding, 2014a). Considering that B3 cells’ phenotypes with retarded growth rates and decreased O_2_-evolving activity under LC conditions were complemented with MBC-3 cells, we concluded that LCIB-Clover in MBC-3 cells was functional in vivo.

Additionally, we also found that the maximum rate of O_2_-evolving activity (V_max_) of B3 cells decreased to 66% and 78% of that of C9 cells when grown in medium aerated with 5% CO_2_ or with 0.04% CO_2_ (Figure  1E). Moreover, the K_0.5_ (CO_2_) value, the CO_2_ concentrations required for half of V_max_, of B3 was 3.8-fold higher than that of the C9 cells when grown with aeration with 5% CO_2_ (Figure  1E). These results suggested that LCIB is necessary for maintaining photosynthetic activity in HC conditions as well as LC conditions.

### LCIB migration to the pyrenoid depends on CO_2_, not on total Ci and ${\text{HCO}}_{3}^{–}$, concentrations in liquid culture medium

**Figure 2:**
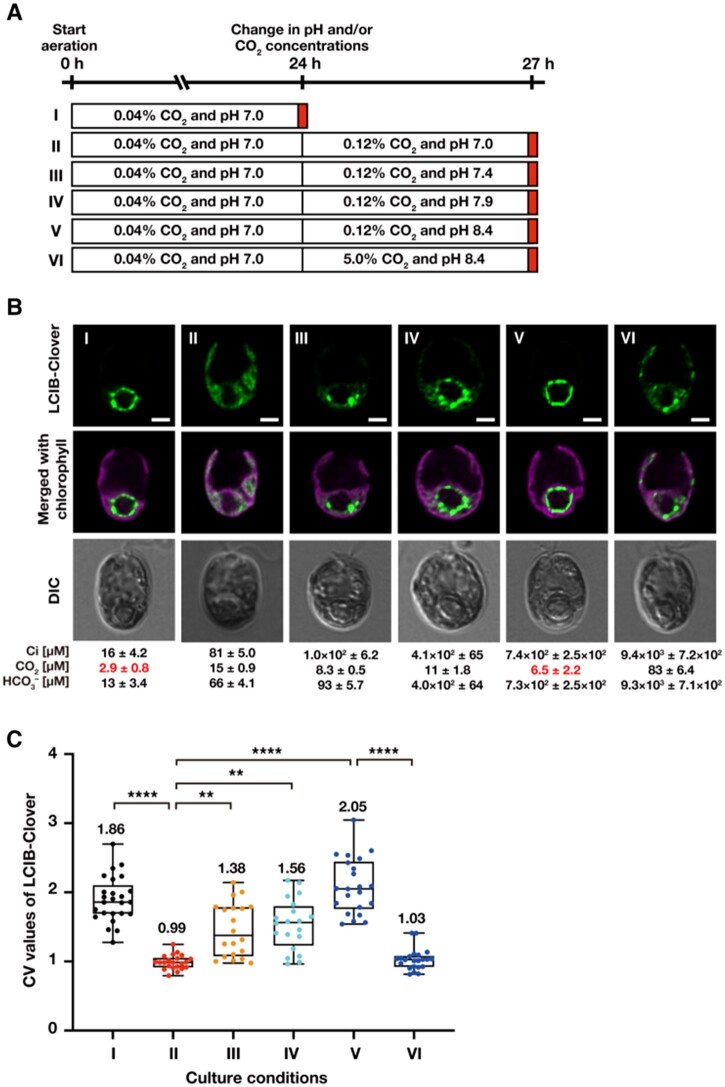
Subcellular localization of LCIB-Clover in different CO_2_ and pH conditions. **A,** Schematic of the liquid culture conditions. MBC-3 cells were cultured in medium aerated with 0.04% CO_2_ at pH 7.0 for 24 h (indicated as I to the left of the white box), which was replaced with fresh medium aerated with 0.12% CO_2_ at pH 7.0 (II), 0.12% CO_2_ at pH 7.4 (III), 0.12% CO_2_ at pH 7.9 (IV), 0.12% CO_2_ at pH 8.4 (V), or 5.0% CO_2_ at pH 8.4 (VI), and cultured for 3 h. Red boxes indicate the time of observation. **B,** Representative LCIB-Clover fluorescence images of MBC-3 cells. Roman numerals correspond to the culture conditions indicated in A. Ci concentrations in the culture medium were measured using gas chromatography, and the calculated CO_2_ and ${\text{HCO}}_{3}^{–}$ concentrations are shown below the images. CO_2_ concentrations shown in red indicate VLC (<7 µM CO_2_) conditions. DIC, differential interference contrast image. Scale bars: 2 μm. **C,** Quantification of localization patterns of LCIB-Clover. Roman numerals correspond to the culture conditions indicated in A. The CV value of the fluorescence intensity was calculated in each cell to quantify LCIB-Clover localization. The median values derived from the analysis of MBC-3 cells are represented with error bars depicting the interquartile range (*n* = 20–24). Dunn’s multiple comparisons test was used to assess the statistical significance of LCIB-Clover localization between the different conditions. ^**^P-value < 0.01; ^****^P-value < 0.0001, Kruskal–Wallis test with Dunn’s multiple comparison.

So far, changes in LCIB localization in liquid culture conditions were observed by varying the CO_2_ concentration (e.g., 5% or 0.04% CO_2_) aerated into the liquid medium. However, because CO_2_ dissolves in water to form dissolved Ci, it was not clear whether LCIB migration depended on CO_2_ or ${\text{HCO}}_{3}^{–}$. To clarify whether LCIB migration to around the pyrenoid depended on either CO_2_ or ${\text{HCO}}_{3}^{–}$ in total Ci in the culture medium, we observed the fluorescence signals of LCIB-Clover in MBC-3 cells with different ratios of ${\text{HCO}}_{3}^{–}$ to CO_2_ concentrations by varying the pH of the medium (Figure  2, A and B). To this end, we measured the actual Ci concentrations ([Ci]) in the culture medium using gas chromatography along with observation and calculated pH-dependent dissolved CO_2_ concentrations ([CO_2_]) and ${\text{HCO}}_{3}^{–}$ concentrations ([${\text{HCO}}_{3}^{–}$]). We also calculated the coefficient of variation (CV) values of LCIB-Clover fluorescence signals to quantify the subcellular localization pattern in the chloroplast (Figure  2C). As shown in previous reports (Nitta et al., 2018; Toyokawa et al., 2020), high- and low-CV values corresponded to aggregation and dispersion patterns of LCIB-Clover in the chloroplast, respectively.

When we cultured MBC-3 cells in medium aerated with 0.04% CO_2_ at pH 7.0 for 24 h, actual [Ci], calculated [CO_2_], and calculated [${\text{HCO}}_{3}^{–}$] were 16, 2.9, and 13 µM, respectively. In this condition, [CO_2_] was in the range of VLC, and LCIB-Clover localized around the pyrenoid as a “ring-like” structure in two-dimensional projection (median CV value 1.86; I in Figure  2B). In three-dimensional reconstructions from Z-stacked images, LCIB-Clover was clustered as several puncta around the pyrenoid (Supplemental Figure S1, A and B and Supplemental Movie S1). Next, when we replaced the medium with those at pH 7.0, 7.4, or 7.9 and cultured with aeration with 0.12% CO_2_ for 3 h, [CO_2_] increased to 8.3–15 µM, which was in the LC range. The CO_2_ concentration of 0.12% was chosen after examining that at which LCIB-Clover dispersed completely at pH 7.0. LCIB-Clover dispersed in the chloroplast at pH 7.0 (median CV value 0.99; II in Figure  2B) or localized in the basal region of the chloroplast at pH 7.4 (median CV value 1.38; III in Figure  2B) and pH 7.9 (median CV value 1.56; IV in Figure  2B). In contrast, when we replaced the medium with that at pH 8.4 with aeration with 0.12% CO_2_ for 3 h, [CO_2_] increased slightly to 6.5 µM but remained in the range of VLC conditions, and LCIB-Clover localized around the pyrenoid (median CV value 2.05; V in Figure  2B) despite the increase in [${\text{HCO}}_{3}^{–}$] from 13 to 730 µM. A CO_2_ concentration of ∼7 µM, at which the LCIB-Clover localization switched from the basal region or dispersed in the chloroplast to the periphery of the pyrenoid, corresponded to the threshold between LC (7–70 µM CO_2_) and VLC (<7 µM CO_2_) estimated from the physiological function of LCIB (Figure  1D). Moreover, considering that LCIB-Clover dispersed in the chloroplast (median CV value 1.03; VI in Figure  2B) when cultured in medium at pH 8.4 and aerated with 5% CO_2_, LCIB migration to around the pyrenoid was not due to the effect of the pH increase itself but the decrease in [CO_2_] in the culture medium to VLC.

### Light, de novo protein synthesis, and photosynthetic electron flow are not required for LCIB relocation

**Figure 3:**
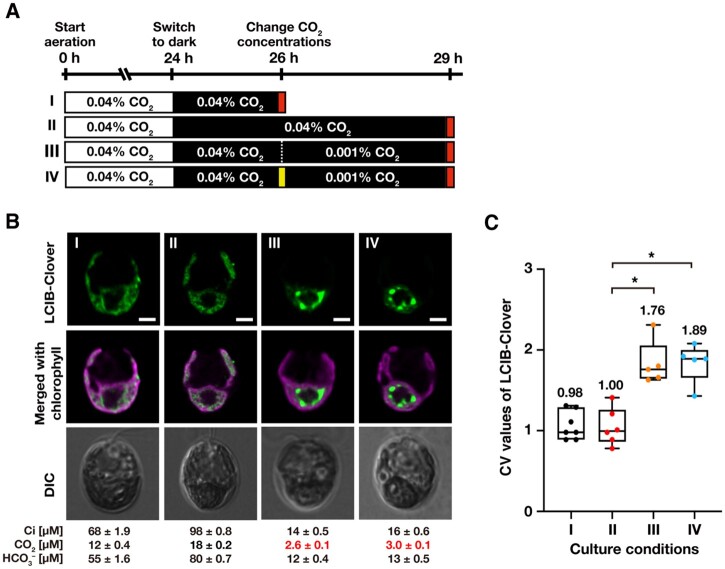
Effect of light and de novo protein synthesis on the relocation of LCIB-Clover. **A**, Schematic of the liquid culture conditions. MBC-3 cells were cultured in medium aerated with 0.04% CO_2_ illuminated at 120 µmol photons m^−2^ s^−1^ (white box) for 24 h were shifted to the dark (black box) for 2 h (indicated as I to the left of the white box) or 5 h (II). After incubation in the dark for 2 h, the concentrations of CO_2_ aeration were switched to 0.001% for 3 h without adding CHX (III) or with the addition of CHX (IV). Red and yellow boxes indicate the time of observation and the addition of CHX, respectively. For all culture conditions, the pH of the medium was 7.0. **B,** Representative LCIB-Clover fluorescence images of MBC-3 cells. Roman numerals correspond to the culture conditions indicated in A. Ci concentrations in the culture medium were measured using gas chromatography, and the calculated CO_2_ and ${\text{HCO}}_{3}^{–}$ concentrations are shown below the images. CO_2_ concentrations shown in red indicate VLC (<7 µM CO_2_) conditions. DIC, differential interference contrast image. Scale bars: 2 μm. **C,** Quantification of localization patterns of LCIB-Clover. Roman numerals correspond to the culture conditions indicated in A. The CV value of the fluorescence intensity was calculated in each cell to quantify LCIB-Clover localization. The median values derived from the analysis of MBC-3 cells are represented with error bars depicting the interquartile range (*n* = 5–7). Dunn’s multiple comparisons test was used to assess the statistical significance of LCIB-Clover localization between the different conditions. ^*^P-value < 0.05, Kruskal–Wallis test with Dunn’s multiple comparison.

**Figure 4:**
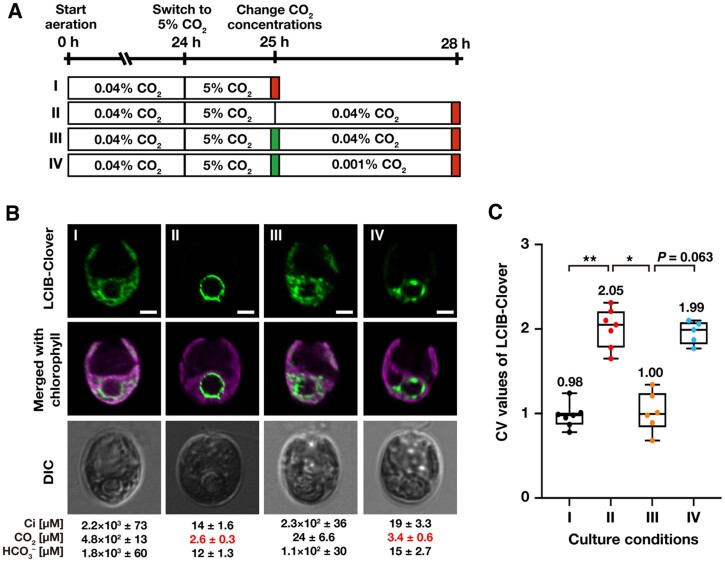
Effect of photosynthetic electron flow on the relocation of LCIB-Clover. **A,** Schematic of the liquid culture conditions. MBC-3 cells were cultured in medium aerated with 0.04% CO_2_ for 24 h, which was replaced with the fresh medium aerated with 5% CO_2_ for 1 h (indicated as I to the left of the white box). After aeration with 5% CO_2_ for 1 h, MBC-3 cells were transferred to fresh medium aerated with 0.04% for 3 h without the addition of DCMU (II), with the addition of DCMU (III), or medium aerated with 0.001% CO_2_ with DCMU (IV). Red and green boxes indicate the time of observation and addition of DCMU, respectively. For all culture conditions, the pH of the medium was 7.0. **B,** Representative LCIB-Clover fluorescence images of MBC-3 cells. Roman numerals correspond to the culture conditions indicated in A. Ci concentrations in the culture medium were measured using gas chromatography, and the calculated CO_2_ and ${\text{HCO}}_{3}^{–}$ concentrations are shown below the images. CO_2_ concentrations shown in red indicate VLC (<7 µM CO_2_) conditions. DIC, differential interference contrast image. Scale bars: 2 μm. **C,** Quantification of localization patterns of LCIB-Clover. Roman numerals correspond to the culture conditions indicated in A. The CV value of the fluorescence intensity was calculated in each cell to quantify LCIB-Clover localization. The median values derived from the analysis of MBC-3 cells are represented with error bars depicting the interquartile range (*n* = 5–7). Dunn's multiple comparisons test was used to assess the statistical significance of LCIB-Clover localization between the different conditions. ^*^P-value < 0.05; ^**^P-value < 0.005, Kruskal–Wallis test with Dunn’s multiple comparison.

In our previous studies, we discussed that LCIB relocation to around the pyrenoid might require light, de novo protein synthesis, or photosynthetic electron flow because LCIB remained dispersed in the chloroplast in the dark or after the addition of cycloheximide (CHX) or 3-(3,4-dichlorophenyl)-1,1-dimethylurea (DCMU; Yamano et al., 2010, 2014). However, because we did not evaluate [CO_2_] in the medium while observing LCIB localization, we could not distinguish whether the dispersion of LCIB was due to the treatments of inhibitors or caused by increased [CO_2_] in the medium. To re-examine the effect of light, de novo protein synthesis, and the photosynthetic electron flow on LCIB relocation, we observed LCIB-Clover in light-to-dark conditions or after treatment with CHX or DCMU along with the measurement of total Ci (Figures  3 and 4).

First, we cultured MBC-3 cells in the light with aeration with 0.04% CO_2_ for 24 h, where CCM was fully induced and [CO_2_] was in the range of VLC (2.9 µM; I in Figure  2B), and transferred them to the dark. After 2 or 5 h in the dark (I and II in Figure  3A), LCIB-Clover dispersed in the chloroplast (median CV value 0.98 and 1.00; I and II in Figure  3, B and C), as shown previously (Yamano et al., 2010). [CO_2_] was increased from 2.9 µM to the range of LC conditions (12–18 µM) due to the cessation of photosynthesis and the activation of respiration in dark conditions. In contrast, when we reduced CO_2_ aeration from 0.04% to 0.001% in the dark (III and IV in Figure  3A), [CO_2_] decreased to the range of VLC conditions (2.6–3.0 µM), and LCIB-Clover relocated to around the pyrenoid even in cells treated with CHX (median CV value 1.76 and 1.89; III and IV in Figure  3, B and C).

Next, we cultured MBC-3 cells in the light with aeration with 0.04% CO_2_ for 24 h, switched the aeration to 5% CO_2_ for 1 h, where LCIB-Clover dispersed in the chloroplast (median CV value 0.98; I in Figure  4, B and C), and then we once again switched aeration from 5% to 0.04% (Figure  4A). At 3 h after transfer to 0.04% CO_2_, LCIB-Clover relocated to around the pyrenoid (median CV value 2.05; II in Figure  4, B and C), as shown previously (Yamano et al., 2010), but adding DCMU inhibited the relocation with the increase in [CO_2_] to the range of LC conditions (24 µM), and LCIB-Clover dispersed in the chloroplast (median CV value 1.00; III in Figure  4, B and C). However, when we switched aeration from 5% to 0.001%, [CO_2_] decreased to the range of VLC conditions (3.4 µM), and LCIB-Clover relocated to around the pyrenoid even in cells treated with DCMU (median CV value 1.99; IV in Figure  4, B and C). Considering these results, light, de novo protein synthesis, and photosynthetic electron flow were not required for LCIB relocation from dispersed in the chloroplast to around the pyrenoid, and the decrease in [CO_2_] in the culture medium was enough for the relocation.

### Illumination of light in VLC conditions is required for LCIB migration to the pyrenoid

**Figure 5:**
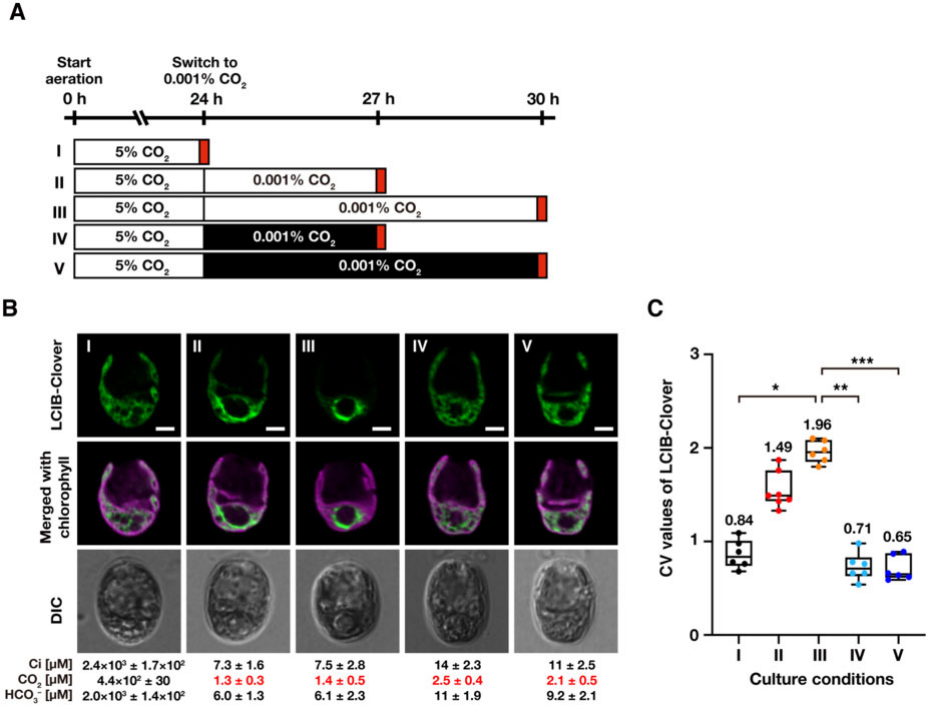
Effect of light on the subcellular migration of LCIB-Clover. **A,** Schematic of the liquid culture conditions. MBC-3 cells were cultured in medium aerated with 5% CO_2_ illuminated at 120 µmol photons m^−2^ s^−1^ (white box) for 24 h (indicated as I to the left of the white box), which was replaced with the fresh medium aerated with 0.001% CO_2_ for 3 h (II) or 6 h (III) in the light or for 3 h (IV) or 6 h (V) in the dark (black box). Red boxes indicate the time of observation. For all culture conditions, the pH of the medium was 7.0. **B,** Representative LCIB-Clover fluorescence images of MBC-3 cells. Roman numerals correspond to the culture conditions indicated in A. Cells were grown in different culture conditions as described in A. Ci concentrations in the culture medium were measured using gas chromatography, and calculated CO_2_ and ${\text{HCO}}_{3}^{–}$ concentrations are shown below the images. CO_2_ concentrations shown in red indicate VLC (<7 µM CO_2_) conditions. DIC, differential interference contrast image. Scale bars: 2 μm. **C,** Quantification of localization patterns of LCIB-Clover. Roman numerals correspond to the culture conditions indicated in A. The CV value of the fluorescence intensity was calculated in each cell to quantify LCIB-Clover localization. The median values derived from the analysis of MBC-3 cells are represented with error bars depicting the interquartile range (*n* = 5–7). Dunn’s multiple comparisons test was used to assess the statistical significance of LCIB-Clover localization between the different conditions. ^*^P-value < 0.05; ^**^P-value < 0.005; ^***^P-value < 0.0005, Kruskal–Wallis test with Dunn’s multiple comparison.

To examine the effect of light on LCIB migration, we cultured MBC-3 cells in the light in medium aerated with 5% CO_2_ for 24 h, where CCM was not induced, switched the aeration to 0.001% CO_2_ in light or dark conditions, and cultured them for 3 or 6 h (Figure  5A). While LCIB-Clover migrated to around the pyrenoid so that it was no longer dispersed in the chloroplast within 6 h in light conditions (median CV value from 0.84 to 1.96; I–III in Figure  5, B and C), LCIB-Clover remained dispersed in the dark (median CV value 0.71 for 3 h and 0.65 for 6 h; IV and V in Figure  5, B and C) despite the decrease in [CO_2_] to the range of VLC conditions (2.1–2.5 µM; IV and V in Figure  5B). These results suggested that light as well as VLC conditions are required for LCIB migration, and other factors induced during CCM induction could be involved in the migration.

### Accumulation of LCIB and LCIC in VLC conditions is required for LCIB migration to the pyrenoid

**Figure 6:**
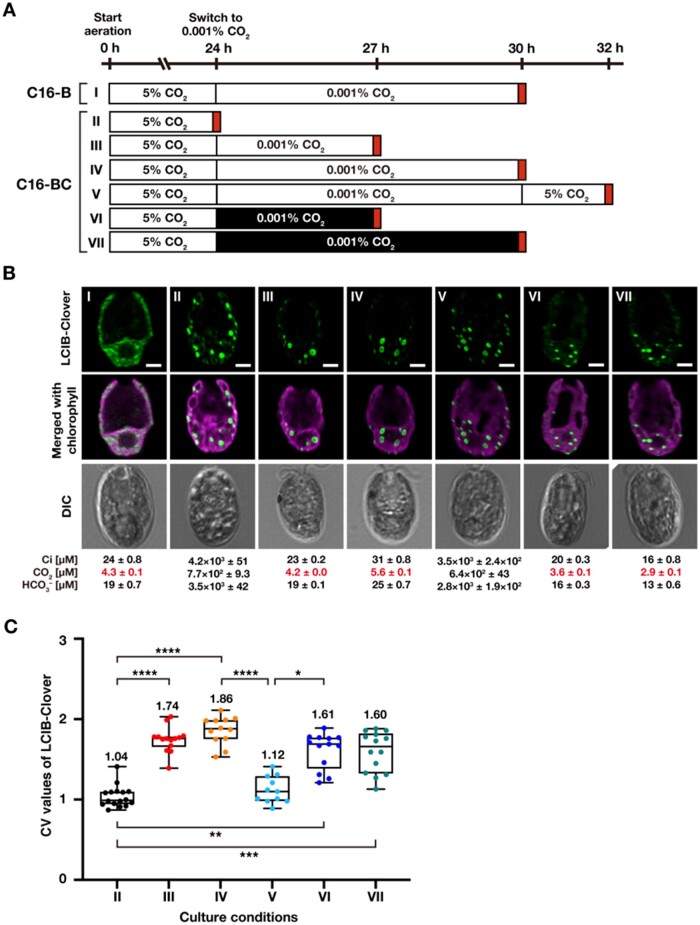
The requirement of LCIC for the LCIB-Clover migration and relocation. **A**, Schematic of the liquid culture conditions. C16-B cells were cultured in medium aerated with 5% CO_2_ illuminated at 120 µmol photons m^−2^ s^−1^ (white box) for 24 h, which was replaced with the fresh medium aerated with 0.001% CO_2_ for 6 h (indicated as I to the left of the white box). C16-BC cells were cultured in medium aerated with 5% CO_2_ for 24 h (II), which was replaced with the fresh medium aerated with 0.001% CO_2_ for 3 h (III) or 6 h (IV) in the light or for 3 h (VI) or 6  h (VII) in the dark (black box). After aeration with 0.001% CO_2_ for 6 h, the concentration of aerated CO_2_ was switched to 5% for 2 h (V). Red boxes indicate the time of observation. For all culture conditions, the pH of the medium is 7.0. **B,** Representative LCIB-Clover fluorescence images of C16-B and C16-BC cells. Roman numerals correspond to the culture conditions indicated in A. Ci concentrations in the culture medium were measured using gas chromatography, and calculated CO_2_ and ${\text{HCO}}_{3}^{–}$ concentrations are shown below the images. CO_2_ concentrations shown in red indicate VLC (<7 µM CO_2_) conditions. DIC, differential interference contrast image. Scale bars: 2 μm. **C,** Quantification of localization patterns of LCIB-Clover. Roman numerals correspond to the culture conditions indicated in A. The CV value of the fluorescence intensity was calculated in each cell to quantify LCIB-Clover localization. The median values derived from the analysis of C16-BC cells are represented with error bars depicting the interquartile range (*n* = 11–16). Dunn’s multiple comparisons test was used to assess the statistical significance of LCIB-Clover localization between the different conditions. ^*^P-value < 0.05; ^**^P-value < 0.01; ^***^P-value < 0.001; ^****^P-value  < 0.0001, Kruskal–Wallis test with Dunn’s multiple comparison.

Because the accumulation of LCIC increases with CCM induction and LCIC interacts with LCIB (Miura et al., 2004; Yamano et al., 2010), we assumed that LCIC is a strong candidate as a factor required for LCIB migration. Thus, to examine the effect of LCIC on LCIB migration, we introduced LCIC and/or LCIB-Clover driven by a constitutive promoter into strain C16 (Figure  6A), in which the CCM was not induced due to a mutation in *CCM1*, a master regulator of the CCM (Fukuzawa et al., 2001). In strain C16-B, where only LCIB-Clover was expressed in strain C16, LCIB-Clover dispersed in the chloroplast even in VLC conditions (I in Figure  6B). On the other hand, in strain C16-BC, where LCIB-Clover and LCIC were overexpressed simultaneously, several speckled structures were observed in the chloroplast in HC conditions (median CV value 1.04; II in Figure  6, B and C). When we cultured C16-BC cells in medium aerated with 0.001% CO_2_ in the light, the speckled structures migrated toward the pyrenoid (median CV value 1.74 for 3 h and 1.86 for 6 h; III and IV in Figure  6, B and C). However, its localization did not uniformly surround the pyrenoid, and 4–7 large speckles with 500–800 nm sizes were observed at the pyrenoid periphery (Supplemental Figure S2, A and B and Supplemental Movie S2). The appearance of the large speckles was not constant; when speckles were larger and fewer, no fluorescence was visible at their cores. After switching the CO_2_ aeration from 0.001% to 5%, these speckles were able to disperse in the chloroplast (median CV value 1.12; V in Figure  6, B and C), and the number was not significantly changed (Supplemental Figure S2, A and B). Although the speckled structures migrated toward the pyrenoid in medium aerated with 0.001% CO_2_ even in the dark (median CV value 1.61 for 3 h and 1.60 for 6 h; VI and VII in Figure  6, B and C), the number of speckles with no fluorescence at their cores were decreased significantly from 4–7 to 1–2 (Supplemental Figure S2, A and B and Supplemental Movie S3). These results suggest that the accumulation of LCIC in VLC conditions is sufficient for LCIB migration, but other factors could be required for the LCIB/LCIC to localize around the pyrenoid uniformly.

## Discussion

In this study, we examined the effects of pH, light availability, photosynthetic electron flow, and protein synthesis on localization changes of LCIB in the algal chloroplast. LCIB migration to around the pyrenoid required the accumulation of LCIC in the *Chlamydomonas* chloroplast, and once LCIB localized around the pyrenoid, reversible changes in LCIB localization were tightly regulated by the external [CO_2_] without any requirement for light, photosynthetic electron flow, or protein accumulation other than LCIC. We also found that LCIB could be responsible for maintaining photosynthetic Ci-affinity even in HC conditions. These results will help to strengthen the current CCM model further.

### Strengthening the current CCM model

The current model of CCM suggests that LCIB is involved in CO_2_ recapturing around the pyrenoid in VLC conditions as well as CO_2_ uptake in LC conditions (Wang and Spalding, 2006; Yamano et al., 2010; Wang and Spalding, 2014a; Toyokawa et al., 2020). From this study, *Chlamydomonas* could actively pump in CO_2_ by the function of LCIB converting the influx of CO_2_ into ${\text{HCO}}_{3}^{–}$ in the chloroplast stroma in HC conditions. Because CA catalyzes the equilibrium reaction between CO_2_ and ${\text{HCO}}_{3}^{–}$, when the ${\text{HCO}}_{3}^{–}$ concentration increases, it is more likely to be converted to CO_2_, resulting in CO_2_ leakage from the cell. A putative mechanism by which LCIB could carry out unidirectional hydration reactions against such a concentration gradient is unknown. One possibility is pH homeostasis in the chloroplast stroma, where the dehydration reaction from ${\text{HCO}}_{3}^{–}$ to CO_2_ is unlikely to occur at pH ∼8. Another possibility is the example of CO_2_-uptake protein (CUP) in the cyanobacterial NDH-1 complex. CUPs have CA activity without a Zn^2+^ binding motif and contribute to CCM with only converting CO_2_ to ${\text{HCO}}_{3}^{–}$ by coupling hydration to an energetically favorable process (Laughlin et al., 2020). The lack of CA activity of LCIB itself, despite its structural feature of β-CA (Jin et al., 2016), may be related to the “pumping CO_2_” function of LCIB, which would work exclusively in the direction of CO_2_ to ${\text{HCO}}_{3}^{–}$.

The different localization patterns of LCIB depending on extracellular CO_2_ levels should reflect multistep CO_2_-regulated acclimation to overcome the difficulty of acquiring Ci in aquatic environments. Such dynamic localization changes are difficult to capture in conventional experiments that deal with cells grown in fixed CO_2_ concentrations, indicating the need to estimate the acclimation state (HC, LC, or VLC) of the cells by constantly measuring and/or calculating the Ci and CO_2_ concentration of the culture medium. Considering that CCM studies need to be done from the ecological and physiological point of view, our finding will strengthen the current CCM model and indicates a more flexible survival strategy for algal cells than previously thought, especially under fluctuating CO_2_ environment.

### CO_2_ acclimation systems in a fluctuating CO_2_ environment

A previous study also reported that [CO_2_] in the medium, but not [${\text{HCO}}_{3}^{–}$], are a critical factor for inducing the activity of ${\text{HCO}}_{3}^{–}$ transport in *Chlamydomonas* and that ${\text{HCO}}_{3}^{–}$ transport activity was fully induced at ∼10 µM of CO_2_ (Bozzo and Colman, 2000). This CO_2_ concentration was close to that (∼7 µM) required to switch LCIB localization patterns in the chloroplast found in this study. Moreover, it was also reported that light is not essential for inducing CA activity and Ci transport activity (Bozzo and Colman, 2000). Considering these results, there are unknown molecular mechanisms to switch the Ci-uptake system between HLA3/LCIA-mediated ${\text{HCO}}_{3}^{–}$ transport along with LCIB-driven CO_2_-recapturing around the pyrenoid in VLC conditions and LCIB-driven CO_2_-uptake in the entire chloroplast in HC and LC conditions to acclimate to fluctuating CO_2_ conditions. Under VLC conditions with a high ${\text{HCO}}_{3}^{–}$/CO_2_ ratio in the chloroplast stroma, it would be important to deactivate the putative CA activity of the LCIB/LCIC complex to prevent CO_2_ leakage due to ${\text{HCO}}_{3}^{–}$ dehydration activity. Recombinant LCIB, LCIC, LCIB-LCIC complex, and native LCIB-LCIC complex do not have CA activity (Jin et al., 2016). Although the stoichiometry of the LCIB/LCIC complex is not yet clear, it cannot yet be ruled out the possibility that the putative CA activity is regulated by the complex formation and dissociation of LCIB and LCIC in response to CO_2_ concentration.

It is not clear to what extent the local CO_2_ concentration fluctuates over short periods in aquatic environments. A previous study using the large diatom *Odontella sinensis* showed that rapid and substantial changes in pH and ${\text{CO}}_{3}^{2–}$ concentrations occurred at the cell surface due to external CA activity and played an important role in photosynthetic Ci-uptake (Chrachri et al., 2018). The CO_2_ concentration at the cell surface may also constantly fluctuate due to respiration and photosynthetic activity in surrounding bacteria and microalgae. Rapid localization changes of LCIB in response to external [CO_2_] without transcription and translation could play an essential role in the flexible acclimation to fluctuating CO_2_ conditions in the aquatic environment. Moreover, because changes in LCIB localization in the chloroplast are easy to observe and are strictly regulated by external [CO_2_], LCIB-Clover used in this study can be used as a molecular marker to estimate the LC and VLC states of the culture medium.

### Mechanism of LCIB migration and relocation

When LCIB-Clover was expressed alone in the *ccm1*-deficient mutant C16, LCIB-Clover was uniformly dispersed throughout the entire stroma. In contrast, when the accumulation of LCIC and LCIB-Clover were overexpressed simultaneously, several speckled structures were observed in the chloroplast. These structures were localized to the pyrenoid periphery in VLC conditions, suggesting that VLC conditions and the interaction between LCIB and LCIC are necessary for the complex to migrate to the pyrenoid. This is consistent with the results that the accumulation of LCIC was also missing in the *lcib* mutant (Figure  1B). So far, there is no solid explanation for why LCIC accumulation could not be detected (or remarkably reduced) in the *lcib* background; there could be negative feedback on LCIC synthesis, or LCIC could be destabilized/degraded due to the loss of LCIB. We previously showed that mRNA expression of *LCIC* was normally induced, but the protein accumulation was markedly reduced in the *LCIB* RNAi lines (Yamano et al., 2010), so the missing of LCIC could occur at the posttranscriptional level. So far, it has been discussed that CCM1 is a master regulator of the CCM in green algae and that CCM1 itself could be a sensor to sense the change in environmental Ci concentrations. However, even in the *ccm1* background, LCIB can respond to the decrease in [CO_2_] by interacting with LCIC and migrate to the pyrenoid periphery, suggesting another CO_2_-sensing mechanism besides CCM1.

The speckle structures of LCIB-Clover located around the pyrenoid had no fluorescence visible at their core and did not localize uniformly around the pyrenoid. These results indicate that other factor(s), such as protein expression and/or posttranslational modification, are necessary to form the ring-like structure around the pyrenoid. Although we previously reported that immunoprecipitated LCIB could be phosphorylated (Yamano et al., 2010), mass spectrometry profile to determine the posttranslational modification under HC, LC, and VLC conditions have not been examined. Considering that LCIB and LCIC are soluble proteins, they could require some form of anchoring to relocalize from the stroma to the pyrenoid periphery. This could be mediated by the pyrenoid tubules, thylakoid membranes penetrating pyrenoid, because the pyrenoid tubules are radially arranged and limited in number, which is consistent with the discontinuous labeling of LCIB. Previous results also showed that LCIB aggregates near the gap in the starch sheath where the thylakoid membranes penetrate the pyrenoid (Yamano et al., 2010).

Recently, it has been examined that three BST1–3 localize to the thylakoid membrane around the pyrenoid and may be involved in ${\text{HCO}}_{3}^{–}$ transport to the lumen of the thylakoid membrane (Mukherjee et al., 2019). Considering that both LCIB and LCIC interact with BST3 (Cre16.g663450), LCIC also interacts with BST1 (Cre16.g662600; Mackinder et al., 2017), and genes encoding BST1–3 are regulated by CCM1 (Yamano et al., 2008), one of BST1–3 may be necessary for LCIB/LCIC to be correctly positioned around the pyrenoid as a ring-like structure. It would be of interest to examine the localization of LCIB and LCIC in mutants missing *BST1–3*. Another possibility is that the pyrenoid structure, including pyrenoid tubules and starch sheaths, did not develop normally in the background of *ccm1* mutants, resulting in abnormal LCIB localization. Because the previous study showed the reduced size of pyrenoid in the mutant strain *ccm1* (Fukuzawa et al., 2001), observation of TEM images of the *ccm1* and C16-BC lines would explain the placement of the speckles around the pyrenoid.

### Toward reconstitution of algal CCMs

There has been research aimed at enhancing the CO_2_-fixing capacity of Rubisco by introducing algal CCMs into the chloroplasts of terrestrial plants. Because the interaction between Rubisco and the Rubisco linker EPYC1 is sufficient to form phase-separated structures and the motif sequences of proteins that bind to Rubisco have been identified (He et al., 2020; Meyer et al., 2020), it should be possible to reconstitute functional pyrenoids by heterologous expression of algal proteins (Atkinson et al., 2020). Furthermore, ${\text{HCO}}_{3}^{–}$ and CO_2_ transporters/channels associated with Ci-uptake have also been identified, and it would also be possible to express and localize them to the correct positions in land plant cells. However, to fully operate the algal CCM in the chloroplasts of terrestrial plants, the starch sheath formed around the pyrenoid and the CAs responsible for CO_2_-uptake and CO_2_-recapturing must also be stably expressed. Notably, the starch sheath morphology itself is essential for the correct localization of LCIB around the pyrenoid (Toyokawa et al., 2020) as well as for the regulation of the pyrenoid number in the chloroplast (Itakura et al., 2019). In terrestrial plants, CO_2_ is taken up into the leaves through the stomata and it diffuses into the chloroplast stroma; therefore, it is essential to properly position CAs in the chloroplast to form ${\text{HCO}}_{3}^{–}$ pools with little CO_2_ leakage. The findings of this study will contribute to understating of the regulation of heterologous expression and localization of algal CAs in the chloroplasts of terrestrial plants.

## Materials and methods

### Strains and culture conditions

The *C.* *reinhardtii* WT strain C9 was provided from IAM Culture Collection at the University of Tokyo and is now available from Microbial Culture Collection at the National Institute for Environmental Studies, Japan, as strain NIES-2235 (alternatively named as CC-5098 in the *Chlamydomonas* Resource Center). *Chlamydomonas* WT strain CC-1690 was obtained from the *Chlamydomonas* Resource Center. The *lcib*-insertion mutant LMJ.RY0402.173287, designated as B1 (Toyokawa et al., 2020), was obtained from the mutant resources of the CLiP (Li et al., 2016).

For the short maintenance of strains, cells were cultured on an agar plate with Tris-acetate-phosphate (TAP) medium and kept at 20°C in dim light (∼1 µmol photons m^−2^ s^−1^). For physiological and biochemical experiments, cells were precultured in 5 mL of liquid TAP medium by vigorous shaking with ∼50 µmol photons m^−2^ s^−1^ and diluted with ∼50 mL of MOPS-P medium, which contained phosphate (620 µM of K_2_HPO_4_ and 412 µM of KH_2_PO_4_), Hutner’s trace elements, and 20 mM MOPS (pH 7.0). Then, cells were cultured by aerating with air containing 5% (v/v) CO_2_ (HC) until the mid-log phase. When changing the concentrations of CO_2_, HC-acclimated cells were centrifuged at 600 *g* for 5 min, resuspended in 50 mL of fresh MOPS-P medium, and cultured with aeration with ordinary air (0.04% CO_2_) or 0.001% CO_2_ for the indicated periods. To create 0.001% CO_2_ gas, ordinary air was passed through 100 mL of 2 N NaOH solution two times while monitoring the CO_2_ concentrations using an infrared CO_2_ analyzer (model LI-7000; LI-COR). Unless indicated otherwise, cells were cultured at 25°C with continuous illumination using white fluorescent light at ∼120 µmol photons m^−2^ s^−1^.

### Genetic crosses

*Chlamydomonas* parental strains with opposite mating types were cultured for 4 d on an agar plate with 1/5 N TAP medium in which the nitrogen (N) source was diluted to one-fifth. Cultured cells on the agar plates were resuspended in 10 mL of N-free gamete-induction medium (Dutcher, 1995) two times and grown for 3 h with illumination at 80 µmol photons m^−2^ s^−1^. A mixture containing 5 mL of each parental strain was left undisturbed at 80 µmol photons m^−2^ s^−1^ for 1 h. The mixture of 500 µL was plated on a 3% TAP agar plate and left overnight with illumination at 80 µmol photons m^−2^ s^−1^, followed by 7 d incubation in the dark. After 7 d, the vegetative cells were removed using a sterile razor blade and the plate was incubated in the light until zygote colonies appeared. Individual zygote colonies were isolated with the use of an inverted microscope (BX41; Olympus, Tokyo, Japan) equipped with a micromanipulator (Narishige, Tokyo, Japan) and a glass needle (Singer Instruments, Somerset, UK).

### Immunoblotting analysis

Cell suspensions were centrifuged at 800 *g* at 4°C for 5 min, resuspended in chilled phosphate-buffered saline (PBS; 137 mM NaCl, 2.7 mM KCl, 10 mM Na_2_HPO_4_, and 2 mM KH_2_OH_4_, pH 7.4) supplemented with a Complete protease inhibitor cocktail (Roche, Basel, Switzerland), and then disrupted for 1 min on ice using a handy sonicator (UR-20P; TOMY). Unbroken cells and cell debris were removed by centrifugation at 17,000 *g* at 4°C for 20 min, and the resulting supernatant was used as a protein crude extract. After measuring and normalizing the concentrations of the extract by Bradford assay, the extract was resuspended in an equal volume of 2 × SDS gel loading buffer (100 mM Tris–HCl, pH 6.8, 200 mM DTT, 4% SDS, 0.2% bromophenol blue (w/v), 20% glycerol (v/v)) and incubated at 65°C for 10 min. After heating, 10 µg of the extract was separated by 10% (w/v) acrylamide SDS–PAGE. After electrophoresis, proteins were electrophoretically transferred to polyvinylidene difluoride membranes (Bio-Rad, Hercules, California, USA). Membranes were blocked with 5% (w/v) non-fat skim milk (Wako) in PBS for 1 h at room temperature. Blocked membranes were washed with PBS containing 0.1% (v/v) Tween-20 (PBS-T) and incubated with the following antibodies in PBS-T for 1 h at 20°C: rabbit anti-LCIB (1:5,000 dilution), rabbit anti-LCIC (1:10,000), rabbit anti-GFP (1:5,000; MBL), and rabbit anti-histone H3 (1:20,000; Abcam, Cambridge, UK). A horseradish peroxidase-conjugated goat anti-rabbit IgG antibody (Life Technologies, Carlsbad, California, USA) was used as a secondary antibody at a dilution of 1:10,000. Immunologically positive signals were visualized using an enhanced chemiluminescence system in accordance with the manufacturer’s instructions (GE Healthcare).

### Spot test analysis

Cells cultured in MOPS-P medium aerated with 5% CO_2_ at the mid-log phase were diluted with fresh MOPS-P medium to OD_730_ of 0.30, 0.15, and 0.07. Then, 3 µL of each cell suspension was spotted onto an agar plate with MOPS-P medium. The plates were kept in a growth chamber supplied with HC (5% CO_2_), LC (0.04% CO_2_), or VLC (0.01% CO_2_) at ∼120 µmol photons m^−2^ s^−1^ for 3–8 d. To create 0.01% CO_2_ gas, ordinary air was passed through 100 mL of 2 N NaOH solution once and by monitoring the CO_2_ concentrations using an infrared CO_2_ analyzer (model LI-7000; LI-COR).

### Measurement of photosynthetic O_2_-evolution

Cultured cells were harvested by centrifugation at 600 *g* for 5 min and resuspended in Ci-depleted 20 mM HEPES-NaOH buffer (pH 7.8) and adjusted to 10–12 μg mL^−1^ chlorophyll. Photosynthetic O_2_-evolution was measured using a Clark-type O_2_ electrode (Hansatech Instruments, King’s Lynn, UK). Cell suspension (1.5 mL) was put into the measurement chamber of an O_2_-electrode and illuminated at 350 µmol photons m^−2^ s^−1^ using a halogen lamp (KTS-150RSV; Tokina, Tokyo, Japan) for 15 min with aeration with N_2_ gas to deplete dissolved Ci from the suspension. The light intensity was increased to 700 µmol photons m^−2^ s^−1^, and then the required volumes (1–10 µL) of NaHCO_3_ stock solution (15, 150, and 750 mM) were injected into the cell suspension every 30 s to yield the desired Ci concentration.

### Measurement of Ci concentrations and cCalculation of CO_2_ and ${\text{HCO}}_{3}^{–}$ concentrations in the culture medium

Cultured cells (1.5 mL) were centrifuged at 17,000 *g* for 1 min to remove the cells, and the supernatant was transferred to a new microtube. Five 10-μL aliquots of the supernatant were directly injected into the gas-stripping column of a gas chromatograph through a hypodermic needle, and total Ci concentrations in the supernatant were measured after methanization in the presence of H_2_ gas by use of a gas chromatograph (GC-8A; Shimadzu, Kyoto, Japan) with a methanizer (MTN-1; Shimadzu). CO_2_ and ${\text{HCO}}_{3}^{–}$ concentrations in the total Ci concentrations were calculated using the Henderson–Hasselbalch equation:

$$\text{pH}=\text{pKa}+{\;\;\text{log}\;}_{10}\left[{\text{HCO}}_{3}{}^{–}\right]/\left[{\text{CO}}_{2}\right]$$

where pKa is the acid dissociation constant of 6.35 and using an ${\text{HCO}}_{3}^{–}$/CO_2_ ratio of 4.46 at pH 7.0, 11.22 at pH 7.4, 35.48 at pH 7.9, and 112.2 at pH 8.4.

### Observation and quantification of subcellular localization of LCIB-Clover

For observation of LCIB-Clover, 2.5 µL of cultured cells were placed between a coverslip and a thin agarose pad. Then, 16-bit digital fluorescence images were acquired with an oil immersion objective lens (HC PL APO 63×/1.40; Leica) using an inverted laser-scanning confocal fluorescence microscope TCS SP8 (Leica) equipped with a sensitive hybrid detector (for detecting LCIB-Clover) and photomultiplier tube detector (for detecting chlorophyll autofluorescence). LCIB-Clover and chlorophyll were excited at 488 nm, and the emissions derived from Clover and chlorophyll were detected at wavelength ranges of 500–520 nm and 648–700 nm, respectively. Image scanning was performed with a pinhole size of 0.5 or 0.6 Airy units, with the z-stack distance of the scan at 150 nm, at a pixel size of 35–45 nm, and with a line scan speed of 200 or 400 Hz. Huygens Essential software (Scientific Volume Imaging BV) was used for the deconvolution of images. The deconvolution of confocal datasets was performed using the point-spread function theoretically calculated from the microscopic parameters attached to the data and a classic maximum likelihood estimation algorithm (settings: maximum iterations: 100; signal-to-noise: 20; quality criterion: 0.05).

To quantify the localization pattern of LCIB-Clover, the CV values of LCIB-Clover fluorescence signals in the chloroplast were calculated. The CV value was defined as the standard deviation (σ) ratio to the mean (μ) of LCIB-Clover fluorescence signals. Using ImageJ (Fiji), a cup-shaped chloroplast area except for the pyrenoid region was defined as the region of interest (ROI) for an individual cell using the chlorophyll channel of confocal fluorescence microscopy images. The σ and µ of LCIB-Clover fluorescence intensity were quantified using the above-defined ROI, and µ/σ was calculated.

## Accession numbers

The accession numbers in the Phytozome database for *Chlamydomonas* genes *LCIB* and *LCIC* are Cre10.g452800 and Cre06.g307500, respectively.

## Supplemental data 

The following materials are available in the online version of this article.

**Supplemental Figure S1.** Z-stack images of confocal sections of LCIB-Clover, merged with the chlorophyll, and DIC in MBC-3 cells grown in VLC conditions.

**Supplemental Figure S2.** Different images of LCIB-Clover in C16-BC cells and quantification of the speckled structures with no fluorescence at their cores.

**Supplemental Table S1.** Photosynthetic parameters of WT and transformants cells.

**Supplemental Movie S1.** Z-stack of confocal sections of LCIB-Clover merged with the chlorophyll in MBC-3 cells grown in VLC conditions.

**Supplemental Movie S2.** Z-stack of confocal sections of LCIB-Clover merged with the chlorophyll in C16-BC cells grown in VLC conditions with light for 6 h.

**Supplemental Movie S3.** Z-stack of confocal sections of LCIB-Clover merged with the chlorophyll in C16-BC cells grown in VLC conditions in the dark for 6 h.

## Supplementary Material

kiab528_Supplementary_Data

## Notes

T.Y. and H.F. conceived and designed the study; C.T. performed most of the experiments; T.Y. performed microscopy observations and data processing; D.S. and T.M. contributed gas chromatography measurements and analyzed the data; T.Y. and H.F. wrote the article, and all authors approved it. T.Y. and H.F. agreed to serve as the authors responsible for contact and ensure communication.

The author responsible for distribution of materials integral to the findings presented in this article in accordance with the policy described in the Instructions for Authors (https://academic.oup.com/plphys/pages/general-instructions) is: Takashi Yamano (tyamano@lif.kyoto-u.ac.jp) and Hideya Fukuzawa (fukuzawa@lif.kyoto-u.ac.jp).
