# Supplementary material for: CO2-dependent migration and relocation of LCIB, a pyrenoid-peripheral protein in Chlamydomonas reinhardtii
Source: Plant Physiol. 2021 Nov 16;188(2):1081–94. doi: 10.1093/plphys/kiab528 (PMC8825250; doi:10.1093/plphys/kiab528)
Supplement: kiab528_Supplementary_Data [file kiab528_supplementary_data.zip › PP2021RA00971R1_Supplemental_Material.pdf]

# Fig. S1

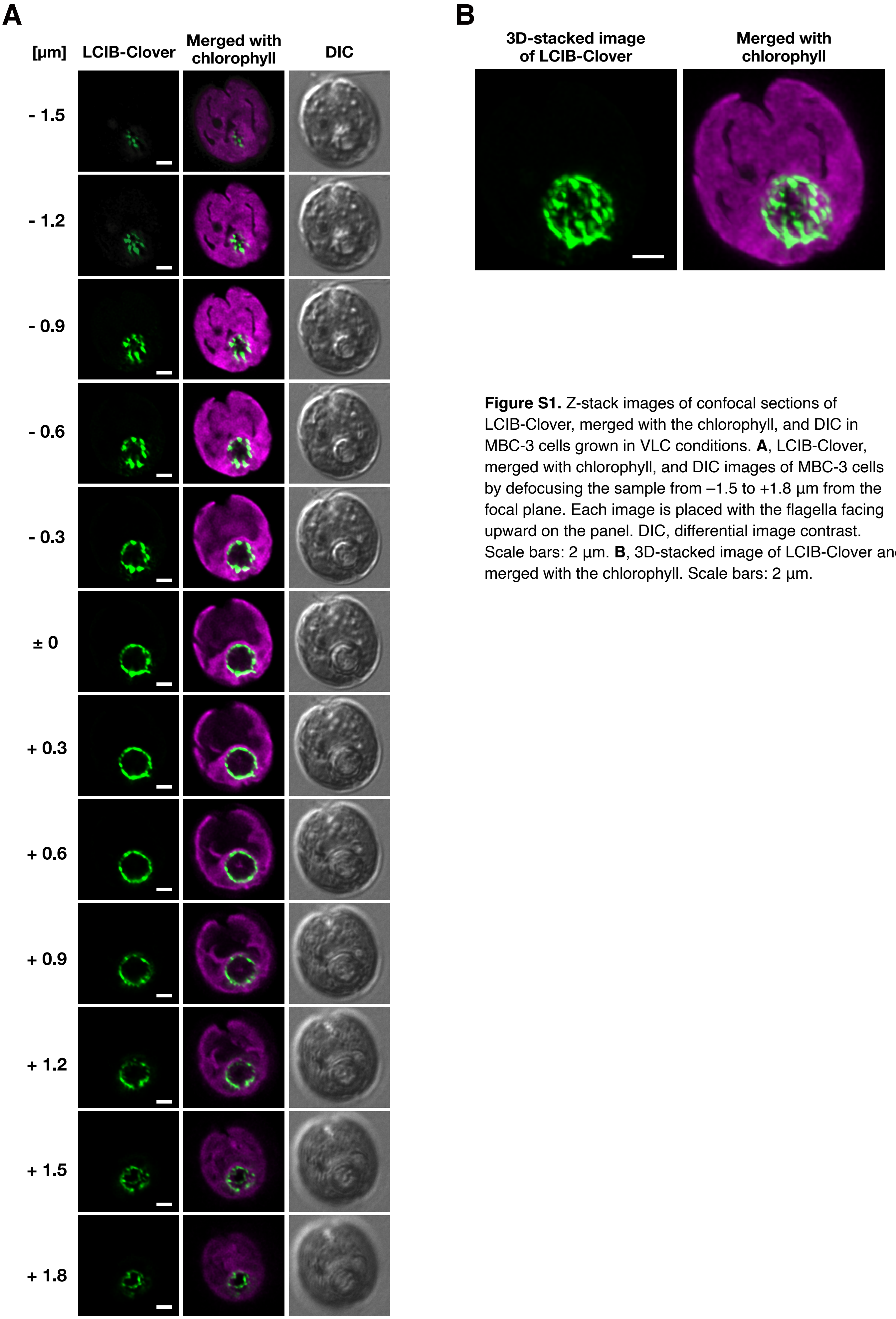


**Figure S1.** Z-stack images of confocal sections of LCIB-Clover, merged with the chlorophyll, and DIC in MBC-3 cells grown in VLC conditions. **A**, LCIB-Clover, merged with chlorophyll, and DIC images of MBC-3 cells by defocusing the sample from –1.5 to +1.8 μm from the focal plane. Each image is placed with the flagella facing upward on the panel. DIC, differential image contrast. Scale bars: 2 μm. **B**, 3D-stacked image of LCIB-Clover and merged with the chlorophyll. Scale bars: 2 μm.

## Fig. S2

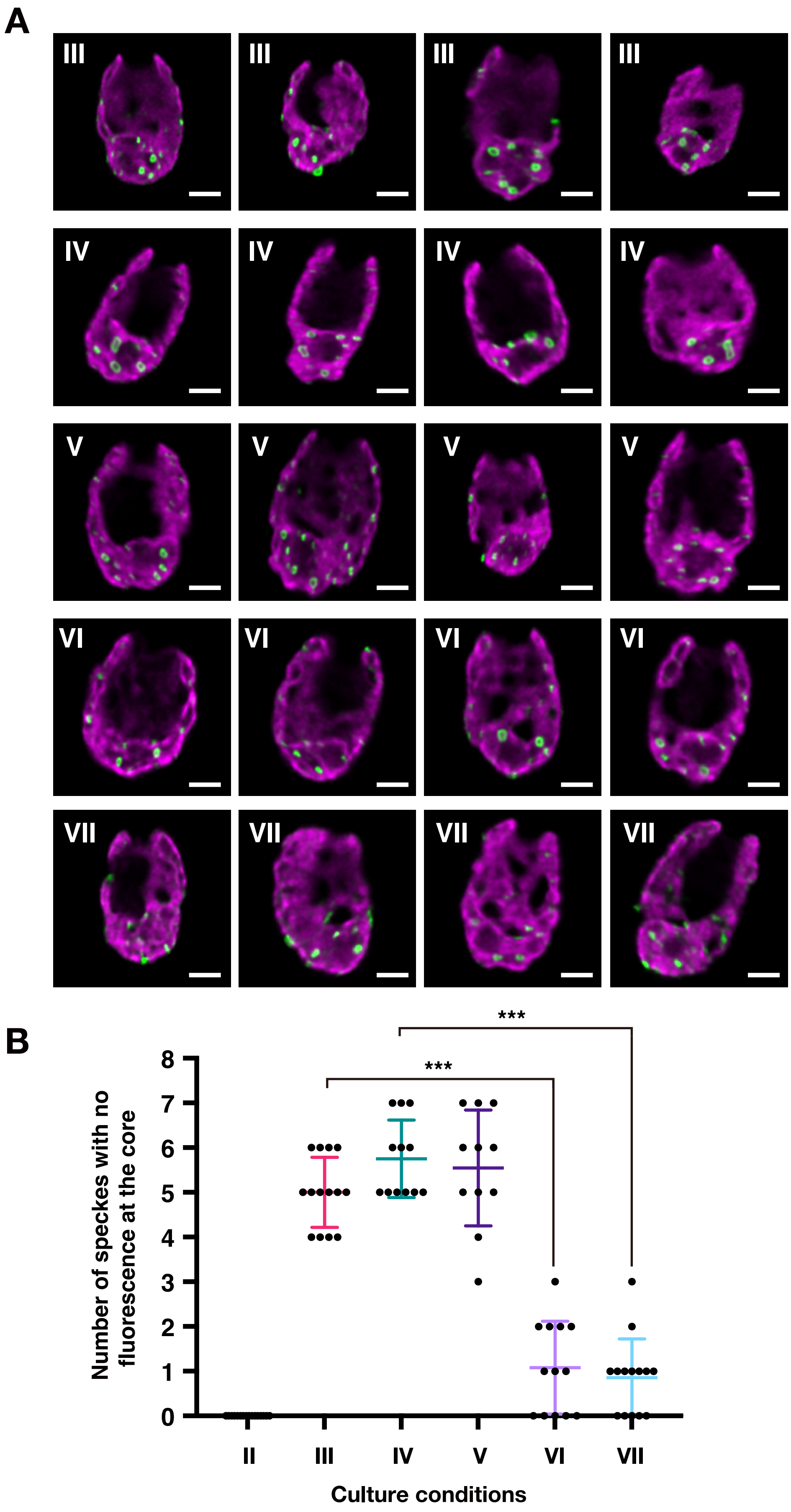


**Figure S2.** Different images of LCIB-Clover in C16-BC cells and quantification of the speckled structures with no fluorescence at their cores. **A**, Different images of LCIB-Clover merged with the chlorophyll of C16-BC cells. Roman numerals correspond to the culture conditions indicated in Fig. 6A. Scale bars: 2 µm. **B**, Quantification of the number of speckles with no fluorescence at the core derived from LCIB-Clover fluorescence signals observed around the pyrenoid. Roman numerals correspond to the culture conditions indicated in Fig. 6A. The median values are represented with error bars depicting the interquartile range (n=11–16). Dunn's multiple comparisons test was used to assess the statistical significance of the number of hollow structures between the different conditions. ***, *P*-value <0.001, Kruskal–Wallis test with Dunn's multiple comparisons.

**Table S1.** Photosynthetic parameters of wild-type and transformant cells.

| Strain name | Growth conditions | $V_{max}$ of $O_2$-evolving activity [μmol $O_2$ $mgChl^{-1}$ $h^{-1}$] | $K_{0.5}$ ($CO_2$) [μM] |
|---|---|---|---|
| C9 | 5% $CO_2$ for 24 h | 191 ± 6 | 13 ± 1.1 |
| | 0.04% $CO_2$ for 12 h | 225 ± 14 | 1.5 ± 0.2 |
| B3 | 5% $CO_2$ for 24 h | 149 ± 4 | 48 ± 2.9 |
| | 0.04% $CO_2$ for 12 h | 147 ± 7 | 1.2 ± 0.1 |
| MBC-3 | 5% $CO_2$ for 24 h | 233 ± 5 | 9.6 ± 2.9 |
| | 0.04% $CO_2$ for 12 h | 211 ± 20 | 1.0 ± 0.1 |

Cells grown in 5% $CO_2$ were shifted to 0.04% $CO_2$ for 12 h or 24 h at 120 μmol photons $m^{-2}$ $s^{-1}$. The data are shown ± standard deviation, which was obtained from more than three independent experiments. $V_{max}$, maximum $O_2$-evolving activity; $K_{0.5}$ ($CO_2$), $CO_2$ concentration required for half of $V_{max}$.
